# ATRP of Methyl Acrylate by Continuous Feeding of Activators Giving Polymers with Predictable End-Group Fidelity

**DOI:** 10.3390/polym11081238

**Published:** 2019-07-26

**Authors:** Yu Wang

**Affiliations:** Department of Chemistry, University of Louisiana at Lafayette, Lafayette, LA 70504, USA; yuwang@louisiana.edu

**Keywords:** atom transfer radical polymerization, ATRP, end-group fidelity, principle of halogen conservation

## Abstract

Atom transfer radical polymerization (ATRP) of methyl acrylate (MA) was carried out by continuous feeding of Cu(I) activators. Typically, the solvent, the monomer, the initiator, and the CuBr_2_/Me_6_TREN deactivator are placed in a Schlenk flask (Me_6_TREN: tris[2-(dimethylamino)ethyl]amine), while the CuBr/Me_6_TREN activator is placed in a gas-tight syringe and added to the reaction mixture at a constant addition rate by using a syringe pump. As expected, the polymerization started when Cu(I) was added and stopped when the addition was completed, and polymers with a narrow molecular weight distribution were obtained. The polymerization rate could be easily adjusted by changing the activator feeding rate. More importantly, the loss of chain end-groups could be precisely predicted since each loss of Br from the chain end resulted in the irreversible oxidation of one Cu(I) to Cu(II). The Cu(I) added to the reaction system may undergo many oxidation/reduction cycles in ATRP equilibrium, but would finally be oxidized to Cu(II) irreversibly. Thus, the loss of chain end-groups simply equals the total amount of Cu(I) added. This technique provides a neat way to synthesize functional polymers with known end-group fidelity.

## 1. Introduction

Atom transfer radical polymerization (ATRP) [1,2,3,4] is a widely-used technique in preparing polymeric materials with various architectures, e.g., block copolymers, star polymers, polymer brushes, and gradient copolymers, for applications in thermoplastic elastomers, nanostructured carbons, surfactants, dispersants, functionalized surfaces, bio-medical materials, etc. [5,6,7]. In ATRP, as well as in any other reversible-deactivation radical polymerization (RDRP) [8], the growth of polymer chains is always accompanied by radical termination. The loss of chain end-groups is not desirable, but cannot be completely avoided [9]. The retention of polymer end-groups is a critical tool for successful chain extension, post polymerization reactions, and controlling material properties [10,11,12,13,14]. For example, in the synthesis of block copolymers, the lower chain end-group fidelity of the macroinitiators resulted in less regular self-assembled nano-structures [15]. Since the end-group fidelity varies greatly depending on the reaction conditions, the batch-to-batch difference may also hinder industrial applications. In the synthesis of cyclic polymers, both α and ω chain end-groups are important [16]. Actually, the narrow molecular weight distribution of the polymers does not guarantee good end-group fidelity [17]. Unfortunately, in many cases, the extent of chain end-group preservation is not only difficult to predict, but also hard to determine. Usually, gel permeation chromatography (GPC) can give a hint about the chain end preservation by comparing the polymers before and after chain extension. However, this cannot provide any quantitative values of the end-group fidelity. More precise characterization techniques are nuclear magnetic resonance spectroscopy (NMR) and matrix-assisted laser desorption ionization time-of-flight mass spectrometry (MALDI-TOF) or electrospray ionization mass spectrometry (ESI-MS) [17,18,19,20]. If the average degree of polymerization is not too high and the polymers have distinguishable chain end-groups in NMR spectroscopy, NMR can give pretty reliable values of end-group fidelity [18,19]. However, the precision decreases with the increasing degree of polymerization since the signal of the chain end-groups diminishes. MALDI-TOF and ESI-MS, on the other hand, provide detailed information of all species with different molecular weights in the sample [17,20]. To get reliable information of the chain end-groups, the molecular weight distribution of the sample must be relatively narrow, and the molecular weight cannot be too high. Besides, the MS spectroscopy of block copolymers is much more complicated. Thus, the chain end-group analysis also becomes more challenging [21]. As a result, methods for precise end-group analysis are not always available. Even if there is a reliable way to determine the end-group fidelity, it is very inconvenient to perform end-group analysis every time after the polymerization and change the reaction conditions if the quality of the product is not good enough. It would be appreciated if a technique can give polymers with known chain end fidelity.

**Scheme 1:**
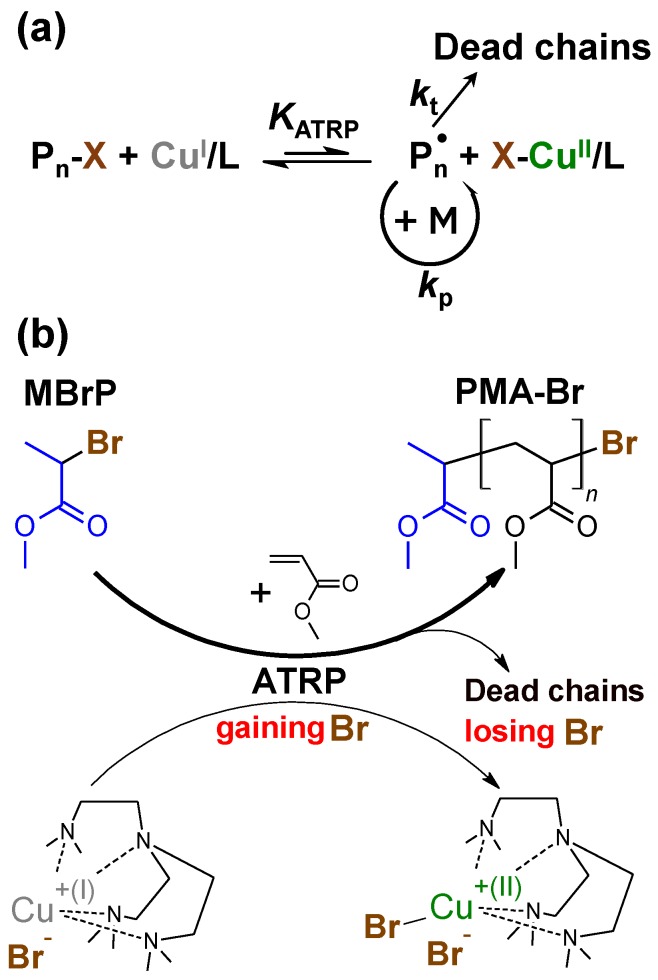
(**a**) General scheme of ATRP equilibrium, where P_*n*_−X is an alkyl halide (macro)initiator, Cu*^I^*/L is the activator complex, P*n* is a (macro)radical, and X−Cu*^II^*/L is the deactivator complex; (**b**) in ATRP of MA by continuous feeding of activators, the loss of chain end Br equals the net irreversible oxidation of Cu(I) to Cu(II), i.e., the principle of halogen conservation.

In ATRP, the loss of chain end-groups results in the transfer of the halogen atom to some other species in the reaction system. Tracking the halogen atom transfer provides a precise way to quantify the loss of chain end-groups [22]. The halogen atom transfer has been used as a tool in the determination of the ATRP equilibrium constant and activation rate coefficient in model systems and during polymerization [23,24,25,26,27]. In normal ATRP, the loss of chain end-groups equals the amount of Cu(I) being irreversibly oxidized to Cu(II) according to the principle of halogen conservation, Scheme 1 and Equation (1):

(1) $${\left[\mathrm{MBrP}\right]}_{0}-\left[\mathrm{PMA}-\mathrm{Br}\right]=\Delta \left[\mathrm{Cu}(\mathrm{II}){\mathrm{Br}}_{2}\right]$$

where ${\left[\mathrm{MBrP}\right]}_{0}$ is the initial concentration of the initiator; [PMA−Br] is the concentration of active polymers with Br chain ends; $\Delta [\mathrm{Cu}(\mathrm{II}){\mathrm{Br}}_{2}]$ is the increase of Cu(II) concentration. In ATRP with activator regeneration processes [3], the chain end loss results in the oxidation of Cu(I) to Cu(II), and then, the Cu(II) is reduced back to Cu(I) chemically or by external regulation, while the halogen atom must transfer to some other reagent in the system, e.g., the reducing agent. In principle, tracking the halogen atom transfer can reveal the amount of chain end-group loss; however, experimentally, this is not easy for either normal ATRP or ATRP with activator regeneration. In this study, ATRP with continuous feeding of the activators was performed. The experimental technique is similar to the previously-reported continuous feeding of reducing agent or radical initiators for ultimate ATRP [28]. However, instead of regenerating Cu(I) by reduction, continuous feeding of the activator could not only provide a useful method to limit the total amount of Cu(I) needed for the polymerization, but also could enable precise prediction of the loss of chain end-groups according to the amount of Cu(I) added to the reaction system.

## 2. Materials and Methods

### 2.1. Materials

Methyl acrylate (MA, 99%, Aldrich, St. Louis, MO, USA) was passed through a column filled with basic alumina prior to use. Methyl 2-bromopropionate (MBrP, 99%, Acros Organics, Morris Plains, NJ, USA), tris[2-(dimethylamino)ethyl]amine (Me_6_TREN, 99+%, Alfa Aesar, Tewksbury, MA, USA), copper(II) bromide (CuBr_2_ 99+%, Acros Organics), copper(II) sulfate pentahydrate (CuSO_4_·5H_2_O, 98+%, Acros Organics), sodium bromide dihydrate (NaBr·2H_2_O, 98+%, Alfa Aesar), sodium sulfite (Na_2_SO_3_, 98+%, Acros Organics), N,N-dimethylformamide (DMF, ACS Certified, Fisher Chemical, Waltham, MA, USA), and acetonitrile (MeCN, HPLC grade, Oakwood Chemical, Estill, SC, USA) were used as received.

### 2.2. Characterization

The molecular weights and the molecular weight distributions of the obtained polymers were characterized by gel permeation chromatography (GPC) using poly(methyl methacrylate) (PMMA) standards for calibration, conducted with a Malvern Viscotek TDA 305-022 system equipped with two T6000M columns and a refractive index detector with THF as an eluent at 30 ${}^{\circ }C$ and at a flow rate of 1 mL/min. The monomer conversion was determined using a Nanalysis 60-MHz Benchtop NMR spectrometer by comparing the signal of the vinyl group and the methyl group. The chain end-group fidelity was characterized using a Varian MR 400-MHz NMR spectrometer by proton NMR 64 scans.

### 2.3. Preparation of CuBr Stock Solution

Three grams of CuSO_4_·5H_2_O and 1.75 g of NaBr·2H_2_O were dissolved in 12.0 mL of water. To the obtained solution, 1.52 g of Na_2_SO_3_ were added over a period of 5 to 10 minutes while stirring. The obtained precipitate of CuBr was collected by filtration, washed twice with water containing a small amount of dissolved sulfurous acid and twice with ethanol, and finally, dried under a vacuum. One-point-zero-nine grams (63% yield) of product were obtained as white powders and stored in a desiccator. A CuBr stock solution was prepared by placing 0.0287 g CuBr in a Schlenk flask, sealed and degassed by allowing N_2_ to flow through it for 1 h. Then, 0.118 mL of Me_6_TREN and 11 mL of MeCN were placed in a round-bottomed flask with a rubber stopper and bubbled with N_2_ for 1 h. Then, 10 mL of the Me_6_TREN/MeCN solution were transferred by using a syringe to the Schlenk flask giving a solution with 0.02 mol/L of CuBr.

### 2.4. General Procedure for Polymerization

A stock solution of 0.0112 g of CuBr_2_ (0.05 mmol) and 0.0230 g of Me_6_TREN (0.1 mmol) in 50 mL of DMF was prepared first. Then, 8.61 g of MA (100 mmol), 0.167 g of MBrP (1 mmol), for a targeted degree of polymerization of 100, and 5 mL of the CuBr_2_ stock solution were added to a 50 mL Schlenk flask. The reaction mixture was degassed by bubbling with N_2_ for 1 h and then placed in an oil bath at a temperature of 60 ${}^{\circ }C$. Two milliliters of the CuBr stock solution were taken into a 2.5 mL Hamilton gas-tight syringe and were added to the reaction mixture over a desired time period at a constant addition rate using a Fisherbrand single-syringe pump. The targeted degree of polymerization was varied by changing the amount of MBrP.

### 2.5. Chain Extension Experiment

The PMA−Br macroinitiator was prepared by the method described above with the targeted degree of polymerization of 50 at 86% monomer conversion, giving the average chain length of 43. The resulting PMA−Br was washed thoroughly with water and then diethyl ether to remove the copper catalyst, the solvent, and the remaining monomer. Then, the white sticky polymer was dried under a vacuum overnight. Then, 3.86 g of PMA−Br macroinitiator (1 mmol), 4.30 g of MA (50 mmol), 2.5 mL of the CuBr_2_ stock solution, and 2.5 mL of DMF were added to a 50 mL Schlenk flask. The reaction mixture was degassed by bubbling with N_2_ for 1 h and then placed in an oil bath at a temperature of 60 ${}^{\circ }C$. Two milliliters of the CuBr stock solution were taken into a 2.5 mL Hamilton gas-tight syringe and were added to the reaction mixture over 2 h at a constant addition rate using a Fisherbrand single-syringe pump.

## 3. Results and Discussion

Normal ATRP of methyl acrylate (MA) with a relatively large amount of Cu(I)/Me_6_TREN (Me_6_TREN: tris[2-(dimethylamino)ethyl]amine), i.e., one equivalent to the initiator, has been reported not to be able to give well-controlled polymerization because of significant early termination and Cu(I)-induced catalytic radical termination [29,30,31]. Normal ATRP of MA with 0.1 equivalent initial Cu(I)/Me_6_TREN resulted in much better control. However, the polymerization was slow and did not reach high monomer conversion [29]. In this study, it was found that polymerization could be well controlled by continuous feeding of Cu(I)/Me_6_TREN. Since Cu(I) has a very short lifetime in the presence of alkyl halide, the concentration of Cu(I) remained very low during the reaction. The polymerization stopped after the addition of Cu(I) was completed, indicating that Cu(I) was oxidized to Cu(II) completely. Thus, the total amount of Cu(I) being used equals the total halogen chain end loss, e.g., 0.05 equivalent Cu(I) to initiator results in $T_{\mathrm{mol}\%}=5\%$, defining the mole percent of chain end halogen loss as $T_{\mathrm{mol}\%}$, Equation (2):

(2) $$T_{\mathrm{mol}\%}=100\times \frac{{\left[\mathrm{MBrP}\right]}_{0}-\left[\mathrm{PMA}-\mathrm{Br}\right]}{{\left[\mathrm{MBrP}\right]}_{0}}\%=100\times \frac{{\left[\mathrm{Cu}(I)\mathrm{Br}\right]}_{\mathrm{total}}}{{\left[\mathrm{MBrP}\right]}_{0}}\%$$

where $T_{\mathrm{mol}\%}$ is the mole percent of chain end halogen loss; ${\left[\mathrm{MBrP}\right]}_{0}$ is the initial concentration of the initiator; [PMA−Br] is the concentration of active polymers with Br chain ends; ${\left[\mathrm{Cu}(I)\mathrm{Br}\right]}_{\mathrm{total}}$ is the total amount of Cu(I) added.

**Figure 1:**
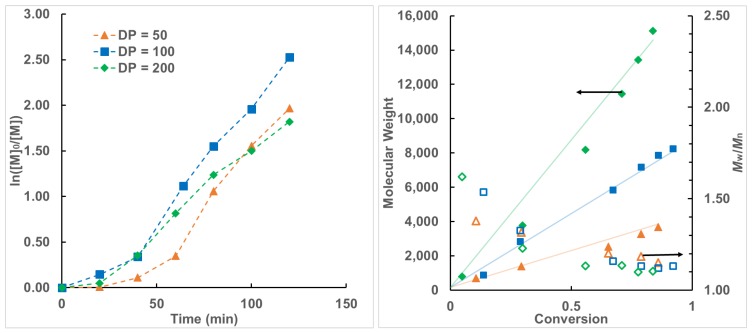
(Left) Kinetic plots of $\ln({\left[M\right]}_{0}/\left[M\right])$ vs. time and (right) plot of number-average molecular weights $M_{n}$ and $M_{w}/M_{n}$ values vs. conversion for ATRP of MA by continuous feeding of Cu(I) activators. ${\left[\mathrm{MA}\right]}_{0}:{\left[{\mathrm{CuBr}}_{2}\right]}_{0}:{\left[{\mathrm{Me}}_{6}\mathrm{TREN}\right]}_{0}=100:0.005:0.01$ at 60 ${}^{\circ }C$ with 100 mmol MA and 2, 1, and 0.5 mmol MBrP, in 5 mL of DMF; 0.04 mmol CuBr/Me_6_TREN in 2 mL of MeCN was added in 2 h.

ATRP of MA was conducted by using methyl 2-bromopropionate (MBrP) as the initiator in dimethylformamide (DMF) as the solvent. In the presence of 50 ppm Cu(II)/Me_6_TREN, the reaction mixture was stirred at 60 ${}^{\circ }C$. A Cu(I)/Me_6_TREN solution in acetonitrile (MeCN) was placed in a gas-tight syringe and was added to the reaction system by a syringe pump at a constant addition rate. The reactions were conducted in well-sealed systems under the protection of N_2_. The Cu(I) solution in the syringe remained colorless throughout the reaction period; see Figure S1 in the Supporting Information. When the reaction was finished, the residual Cu(I) solution was exposed to air and changed to a green color immediately; see Figure S2 in the Supporting Information. Well-controlled polymerization was observed with different targeted degrees of polymerization ($DP_{T}$), which equal the initial molar ratio between the monomer and the initiator; see Figure 1. In all three reactions, the amount of monomer was fixed at 100 mmol. Various amounts of MBrP, i.e., 2 mmol, 1 mmol, and 0.5 mmol, were used for $DP_{T}=50,100,200$, respectively. For all three reactions, 0.04 mmol Cu(I)/Me_6_TREN was added in 2 h, and the polymerizations all reached over 80% monomer conversions. No further monomer conversion was observed after Cu(I) addition was completed. The molecular weights of the poly(methyl acrylate) (PMA) agreed with the theoretical values, and narrow molecular weight distributions, i.e., $M_{w}/M_{n}<1.15$, were observed. According to the Cu(I) to initiator ratio, the mole percent of the loss of chain end-groups was $T_{\mathrm{mol}\%}=2\%,4\%,8\%$ for $DP_{T}=50,100,200$, respectively. One polymerization with $DP_{T}=100$ without initially added Cu(II) was also conducted under identical conditions. Broader molecular weight distributions were observed; see Figure S3 in the Supporting Information. The final monomer conversion reached 95%, giving polymers with $M_{n}=8700$ and $M_{w}/M_{n}=1.31$. Since the Cu(I) to initiator ratio was also 0.04, the $T_{\mathrm{mol}\%}$ should be 4% (3% according to ^1^H NMR) regardless of the broad molecular weight distribution.

In all three reactions, the polymerization rate was relatively slow at the beginning and accelerated after a certain time. The possible explanation is that termination of small molecular weight radicals is much faster than that of polymeric radials [32]. Thus, the radical concentration increased as the polymer chains were growing. As expected, the induction period seemed the longest for $DP_{T}=50$ because the molecular weight increase was the slowest. In addition, a trace amount of oxygen and the inhibitor residual might also contribute to this phenomenon and affect the overall polymerization rate and final monomer conversion.

**Figure 2:**
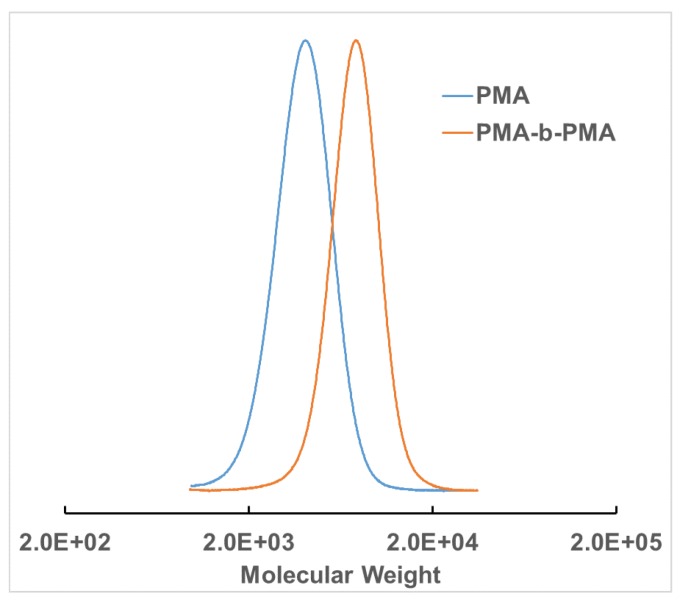
GPC curves of the chain extension experiment with PMA_4_3−Br as the macroinitiator. ${\left[\mathrm{MA}\right]}_{0}:{\left[{\mathrm{PMA}}_{43}-\mathrm{Br}\right]}_{0}:{\left[{\mathrm{CuBr}}_{2}\right]}_{0}:{\left[{\mathrm{Me}}_{6}\mathrm{TREN}\right]}_{0}=50:1:0.0025:0.005$ at 60 ${}^{\circ }C$ with 50 mmol MA in 5 mL of DMF; 0.04 mmol CuBr/Me_6_TREN in 2 mL MeCN was added in 2 h. Before chain extension, $M_{n}=3700$, $M_{w}/M_{n}=1.15$; after chain extension, $M_{n}=7300$, $M_{w}/M_{n}=1.09$.

The chain extension experiment was performed with the PMA−Br produced in the previous section with $DP_{T}=50$. The final monomer conversion was 87%, giving average DP=43. A molecular weight increase was observed in 2 h with 0.04 equivalent Cu(I)/Me_6_TREN to the macroinitiator, which means only an additional 4% chain end Br loss was induced; see Figure 2. According to GPC analysis, the number average molecular weight increased from 3700 to 7300, while the $M_{w}/M_{n}$ value changed from 1.15 to 1.09. In this chain extension experiment, the induction period was not observed (Figure S4 in the Supporting Information), confirming that the fast termination of small molecular radicals was the main reason causing the slow polymerization at the beginning.

**Figure 3:**
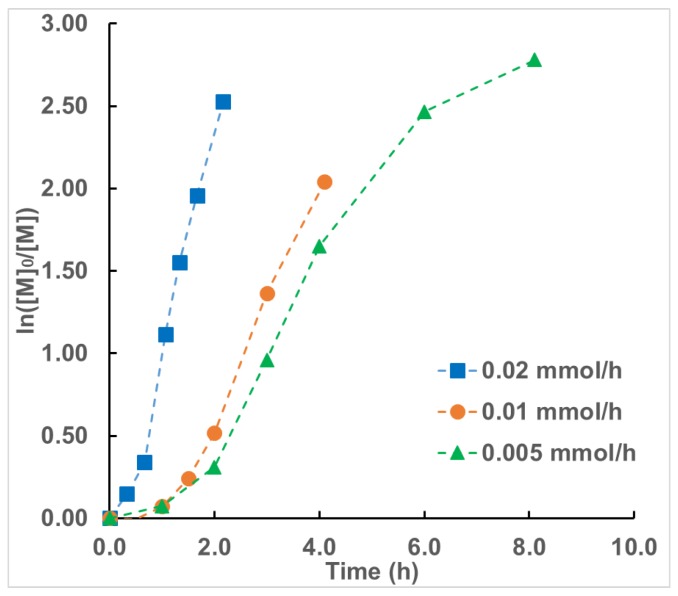
Kinetic plots of $\ln({\left[M\right]}_{0}/\left[M\right])$ vs. time for ATRP of MA by continuous feeding of Cu(I) activators. ${\left[\mathrm{MA}\right]}_{0}:{\left[\mathrm{MBrP}\right]}_{0}:{\left[{\mathrm{CuBr}}_{2}\right]}_{0}:{\left[{\mathrm{Me}}_{6}\mathrm{TREN}\right]}_{0}=100:1:0.005:0.01$ at 60 ${}^{\circ }C$ with 100 mmol MA in 5 mL of DMF; 0.04 mmol CuBr/Me_6_TREN in 2 mL MeCN was added in 2 h, 4 h, and 8 h, respectively.

The polymerization rate could be adjusted by changing the addition rate of Cu(I); see Figure 3. As expected, slower addition of Cu(I) resulted in slower polymerization. Slower polymerization is supposed to give higher monomer conversion at the same level of chain end-group loss. However, this trend was not clear in the experiments. When half Cu(I) was added, the conversion reached 67%, 40% and 81% for an addition rate of 0.02 mmol/h, 0.01 mmol/h, and 0.005 mmol/h, respectively. Though the polymerizations were carried out very carefully, a trace amount of oxygen leakage was still possible and might have affected the polymerization rate. The presence of a small amount of oxygen and residual inhibitor might cause some Cu(I) consumption. Thus, the amount of reactive Cu(I) would be slightly lower than the amount added. The loss of end-groups should also be a little less than the calculated values. Despite the possible imperfection of the experiments, all three polymerizations reached >85% monomer conversion and resulted in polymers with molecular weights agreeing with the theoretical values and $M_{w}/M_{n}<1.15$.

**Figure 4:**
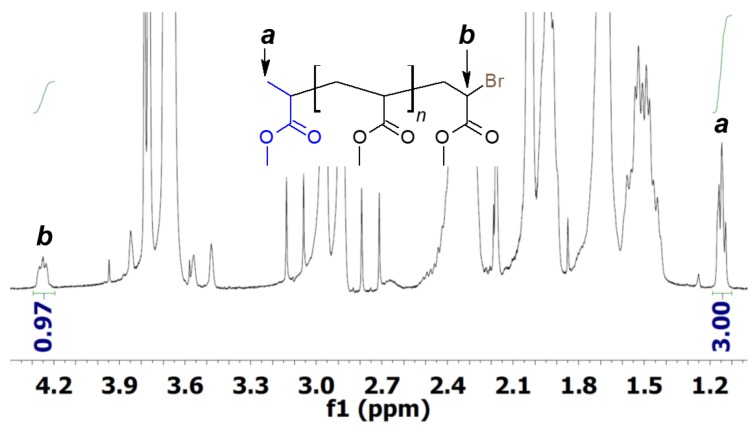
Determination of chain end-group fidelity by ^1^H NMR for PMA−Br obtained via ATRP of MA by continuous feeding of Cu(I) activators. ${\left[\mathrm{MA}\right]}_{0}:{\left[\mathrm{MBrP}\right]}_{0}:{\left[{\mathrm{CuBr}}_{2}\right]}_{0}:{\left[{\mathrm{Me}}_{6}\mathrm{TREN}\right]}_{0}=50:1:0.0025:0.005$ at 60 ${}^{\circ }C$ with 100 mmol MA in 5 mL of DMF; 0.04 mmol CuBr/Me_6_TREN in 2 mL MeCN was added in 2 h. The conversion of monomers reached 86%, giving polymers with $M_{n}=3700$ and $M_{w}/M_{n}=1.15$.

Chain end-group fidelity was determined by ^1^H NMR to compare with the values calculated according to halogen conservation. The protons of the CH_3_ from the initiator and the proton of CH located in the α-position of the bromine chain end were compared; see Figure 4. Without chain end Br loss, the integration area ratio should be 3:1. When there is chain end Br loss, the mole percent of end-group loss can be calculated by Equation (3):

(3) $$T_{\mathrm{mol}\%}^{\mathrm{NMR}}=100\times \left(1-\frac{H_{b}}{H_{a}/3}\right)\%$$

where $T_{\mathrm{mol}\%}^{\mathrm{NMR}}$ is the mole percent of chain end halogen loss calculated by ^1^H NMR analysis; H*_a_* is the integration area of the protons of the CH_3_ from the initiator; H_*b*_ is the integration area of the proton of CH located in the α-position of the bromine chain end. For the example shown in Figure 4, the ^1^H NMR revealed that the chain end-group fidelity was very good, giving $T_{\mathrm{mol}\%}^{\mathrm{NMR}}=3\%$, while the Cu(I) to initiator ratio was 0.02, indicating that actually 2% chain end loss should have taken place. Since the signals of end-groups were very weak in ^1^H NMR, the calculated $T_{\mathrm{mol}\%}^{\mathrm{NMR}}$ values were subject to have certain errors. The calculated values of $T_{\mathrm{mol}\%}$ based on halogen conservation seemed to be more reliable.

The results of the experiments are summarized in Table 1. The polymerizations with different DP or different Cu(I) addition rates, as well as in the chain extension experiment all resulted in well-controlled polymerizations giving PMA with expected molecular weights and narrow molecular weight distributions. The mole percent of end-group loss can be calculated based on the principle of halogen conservation. Thus, the potential loss of chain end-groups was known before the polymerization started. The ^1^H NMR analysis revealed that in these experiments, the end-group fidelity was indeed excellent. However, the $T_{\mathrm{mol}\%}$ values calculated by ^1^H NMR had more significant errors. In the last entry in Table 1, the $T_{\mathrm{mol}\%}^{\mathrm{NMR}}$ was determined to be ∼0%, which could not be true since the conversion from Cu(I) to Cu(II) must have consumed some chain end halogen. Notice that all ^1^H NMR analyses were performed with the reaction mixtures without purification of the polymers, while the macroinitiator used for the chain extension was purified by washing with water and diethyl ether. The purification process should have removed small molecular terminated chains, which would result in a lower mole percent of dead chains than the calculated $T_{\mathrm{mol}\%}^{\mathrm{HC}}$.

The propagation rate coefficient of MA at 60 ${}^{\circ }C$ is 33100 M^−1^s^−1^ [33]. Knowing the monomer conversion and reaction time, one can calculate the average radical concentration. Knowing the overall extent of “termination”, it is possible to calculate the apparent termination rate coefficients, $k_{t}^{\mathrm{app}}$, Equation (4):

(4) $$k_{t}^{\mathrm{app}}=\frac{k_{p}^{2}t{\left[\mathrm{Cu}(I)\mathrm{Br}\right]}_{\mathrm{total}}}{2\ln^{2}\frac{{\left[M\right]}_{0}}{\left[M\right]}}$$

where $k_{t}^{\mathrm{app}}$ is the apparent termination rate coefficient; $k_{p}$ is the propagation rate coefficient; [Cu(I)Br]_total_ is the total amount of Cu(I) added; ${\left[M\right]}_{0}$ and [M] are the initial concentration of monomers and the concentration at a given time. It was found that in all these reactions, the $k_{t}^{\mathrm{app}}$ values were greater than $10^{9}$, which was significantly higher than the average termination rate coefficient in conventional radical polymerization [34]. Though these values could not be considered as precise kinetic parameters, they provide some hints that the catalytic radical termination seemed to contribute significantly to these reactions. Since the concentration of Cu(I) was very low during the polymerizations, the rate-determining step of the catalytic radical termination might be also quite different from the previous report [31]. The technique of ATRP by continuous feeding of Cu(I) may provide a tool to reveal more details of the mechanism of Cu(I)-induced catalytic radical termination and to examine what is a better ligand for ATRP, which could lead to less catalytic radical termination [35,36].

## 4. Conclusions

In summary, ATRP by continuous feeding of activators provided a way to synthesize polymers with precisely-controlled end-group fidelity. When using highly-active ATRP catalyst, i.e., CuBr/Me_6_TREN, the lifetime of Cu(I) is very short. The continuous feeding technique avoided the quick consumption of all Cu(I) activators and helped to maintain a proper concentration of the radicals. The polymerization rate could be adjusted by changing the activator addition rate. The end-group fidelity was known at the time when the polymerization started, which depended on the activator to initiator ratio. The results also gave hints about the extent of Cu(I)-catalyzed termination in the polymerization and provided a new aspect to identify better ligands for ATRP, which would give lower $k_{t}^{\mathrm{app}}$ values. The application of ATRP by continuous feeding of activators to other monomers with different ligands is under investigation and will be reported separately.

## Figures and Tables

**Table 1:** ATRP of MA by continuous feeding of activators.

${\mathrm{DP}}_{T}$	time	conv	$M_{n}^{\mathrm{th}}$	$M_{n}^{\mathrm{GPC}}$	$M_{w}/M_{n}$	$k_{t}^{\mathrm{app}}/10^{8}$ ^*a*^	$T_{\mathrm{mol}\%}^{\mathrm{HC}}$ ^*b*^	$T_{\mathrm{mol}\%}^{\mathrm{NMR}}$ ^*c*^
50	2 h	86%	3900	3700	1.15	26	2%	3%
100	2 h	92%	8100	8200	1.13	16	4%	5%
100	4 h	87%	7700	7900	1.10	49	4%	6%
100	8 h	94%	8200	9600	1.10	52	4%	4%
200	2 h	84%	14,600	15,100	1.10	30	8%	6%
50 ^*d*^	2 h	73%	6500	7300	1.09	60	6%	∼0%

*a* Average apparent radical termination rate coefficient calculated by Equation (4). *b* $T_{\mathrm{mol}\%}$ calculated by halogen conservation, Equation (2). *c* $T_{\mathrm{mol}\%}$ determined by ^1^H NMR. *d* Chain extension from PMA_4_3−Br.

## Supplementary Materials

Supplementary materials are available online at https://www.mdpi.com/2073-4360/11/8/1238/s1. Figure S1: Experimental setup for ATRP by continuous feeding of activators, Figure S2: Color of the Cu(I) stock solution and when it was exposed to air after the reaction, Figure S3: GPC curves for ATRP of MA by continuous feeding of Cu(I) activators at 60 ${}^{\circ }C$ with 100 mmol MA in 5 mL of DMF; 0.04 mmol CuBr/Me_6_TREN in 2 mL of MeCN was added in 2 h (a) with initially-added Cu(II), ${\left[\mathrm{MA}\right]}_{0}:{\left[\mathrm{MBrP}\right]}_{0}:{\left[{\mathrm{CuBr}}_{2}\right]}_{0}:{\left[{\mathrm{Me}}_{6}\mathrm{TREN}\right]}_{0}=100:1:0.005:0.01$, and (b) without initially-added Cu(II), ${\left[\mathrm{MA}\right]}_{0}:{\left[\mathrm{MBrP}\right]}_{0}=100:1$., Figure S4: Kinetic plots of $\ln({\left[M\right]}_{0}/\left[M\right])$ vs. time for ATRP of MA by continuous feeding of Cu(I) activators for the chain extension with PMA_4_3−Br as the macroinitiator. ${\left[\mathrm{MA}\right]}_{0}:{\left[{\mathrm{PMA}}_{43}-\mathrm{Br}\right]}_{0}:{\left[{\mathrm{CuBr}}_{2}\right]}_{0}:{\left[{\mathrm{Me}}_{6}\mathrm{TREN}\right]}_{0}=50:1:0.0025:0.005$ at 60 ${}^{\circ }C$ with 50 mmol MA in 5 mL of DMF; 0.04 mmol CuBr/Me_6_TREN in 2 mL of MeCN was added in 2 h.

## References

[1] Matyjaszewski K., Xia J. (2001). Atom Transfer Radical Polymerization. Chem. Rev..

[2] Tsarevsky N.V., Matyjaszewski K. (2007). “Green” Atom Transfer Radical Polymerization: From Process Design to Preparation of Well-Defined Environmentally Friendly Polymeric Materials. Chem. Rev..

[3] Pan X., Fantin M., Yuan F., Matyjaszewski K. (2018). Externally controlled atom transfer radical polymerization. Chem. Soc. Rev..

[4] Fang C., Fantin M., Pan X., de Fiebre K., Coote M.L., Matyjaszewski K., Liu P. (2019). Mechanistically Guided Predictive Models for Ligand and Initiator Effects in Copper-Catalyzed Atom Transfer Radical Polymerization (Cu-ATRP). J. Am. Chem. Soc..

[5] Coessens V., Pintauer T., Matyjaszewski K. (2001). Functional polymers by atom transfer radical polymerization. Prog. Polym. Sci..

[6] Matyjaszewski K., Tsarevsky N.V. (2014). Macromolecular Engineering by Atom Transfer Radical Polymerization. J. Am. Chem. Soc..

[7] Matyjaszewski K. (2018). Advanced Materials by Atom Transfer Radical Polymerization. Adv. Mater..

[8] Braunecker W.A., Matyjaszewski K. (2007). Controlled/living radical polymerization: Features, developments, and perspectives. Prog. Polym. Sci..

[9] Zhong M., Matyjaszewski K. (2011). How Fast Can a CRP Be Conducted with Preserved Chain End Functionality?. Macromolecules.

[10] Lunn D.J., Discekici E.H., Read de Alaniz J., Gutekunst W.R., Hawker C.J. (2017). Established and emerging strategies for polymer chain-end modification. J. Polym. Sci. Part A Polym. Chem..

[11] Anastasaki A., Willenbacher J., Fleischmann C., Gutekunst W.R., Hawker C.J. (2017). End group modification of poly(acrylates) obtained via ATRP: A user guide. Polym. Chem..

[12] Gutekunst W.R., Anastasaki A., Lunn D.J., Truong N.P., Whitfield R., Jones G.R., Treat N.J., Abdilla A., Barton B.E., Clark P.G. (2017). Practical Chain-End Reduction of Polymers Obtained with ATRP. Macromol. Chem. Phys..

[13] Whitfield R., Anastasaki A., Truong N.P., Wilson P., Kempe K., Burns J.A., Davis T.P., Haddleton D.M. (2016). Well-Defined PDMAEA Stars via Cu(0)-Mediated Reversible Deactivation Radical Polymerization. Macromolecules.

[14] Barbon S.M., Rolland M., Anastasaki A., Truong N.P., Schulze M.W., Bates C.M., Hawker C.J. (2018). Macrocyclic Side-Chain Monomers for Photoinduced ATRP: Synthesis and Properties versus Long-Chain Linear Isomers. Macromolecules.

[15] Pospiech D., Jehnichen D., Eckstein K., Scheibe P., Komber H., Sahre K., Janke A., Reuter U., Häußler L., Schellkopf L. (2017). Semifluorinated PMMA Block Copolymers: Synthesis, Nanostructure, and Thin Film Properties. Macromol. Chem. Phys..

[16] Elupula R., Oh J., Haque F.M., Chang T., Grayson S.M. (2016). Determining the Origins of Impurities during Azide–Alkyne Click Cyclization of Polystyrene. Macromolecules.

[17] Oh J., Kuk J., Lee T., Ye J., Paik H.J., Lee H.W., Chang T. (2017). Molecular Weight Distribution of Living Chains in Polystyrene Prepared by Atom Transfer Radical Polymerization. ACS Macro Lett..

[18] Nguyen N.H., Levere M.E., Kulis J., Monteiro M.J., Percec V. (2012). Analysis of the Cu(0)-Catalyzed Polymerization of Methyl Acrylate in Disproportionating and Nondisproportionating Solvents. Macromolecules.

[19] Lligadas G., Rosen B.M., Monteiro M.J., Percec V. (2008). Solvent Choice Differentiates SET-LRP and Cu-Mediated Radical Polymerization with Non-First-Order Kinetics. Macromolecules.

[20] Nyström F., Soeriyadi A.H., Boyer C., Zetterlund P.B., Whittaker M.R. (2011). End-group fidelity of copper(0)-meditated radical polymerization at high monomer conversion: An ESI-MS investigation. J. Polym. Sci. Part A Polym. Chem..

[21] Town J.S., Jones G.R., Haddleton D.M. (2018). MALDI-LID-ToF/ToF analysis of statistical and diblock polyacrylate copolymers. Polym. Chem..

[22] Wang Y., Zhong M., Zhang Y., Magenau A.J.D., Matyjaszewski K. (2012). Halogen Conservation in Atom Transfer Radical Polymerization. Macromolecules.

[23] Tang W., Tsarevsky N.V., Matyjaszewski K. (2006). Determination of Equilibrium Constants for Atom Transfer Radical Polymerization. J. Am. Chem. Soc..

[24] Tang W., Matyjaszewski K. (2006). Effect of Ligand Structure on Activation Rate Constants in ATRP. Macromolecules.

[25] Tang W., Matyjaszewski K. (2007). Effects of Initiator Structure on Activation Rate Constants in ATRP. Macromolecules.

[26] Tang W., Kwak Y., Braunecker W., Tsarevsky N.V., Coote M.L., Matyjaszewski K. (2008). Understanding Atom Transfer Radical Polymerization: Effect of Ligand and Initiator Structures on the Equilibrium Constants. J. Am. Chem. Soc..

[27] Wang Y., Kwak Y., Buback J., Buback M., Matyjaszewski K. (2012). Determination of ATRP equilibrium constants under polymerization conditions. ACS Macro Lett..

[28] Jakubowski W. (2012). Adapting Atom Transfer Radical Polymerization to Industrial Scale Production: The Ultimate ATRP SM Technology.

[29] Matyjaszewski K., Tsarevsky N.V., Braunecker W.A., Dong H., Huang J., Jakubowski W., Kwak Y., Nicolay R., Tang W., Yoon J.A. (2007). Role of Cu 0 in Controlled/“Living” Radical Polymerization. Macromolecules.

[30] Schröder K., Konkolewicz D., Poli R., Matyjaszewski K. (2012). Formation and Possible Reactions of Organometallic Intermediates with Active Copper(I) Catalysts in ATRP. Organometallics.

[31] Wang Y., Soerensen N., Zhong M., Schroeder H., Buback M., Matyjaszewski K. (2013). Improving the “Livingness” of ATRP by Reducing Cu Catalyst Concentration. Macromolecules.

[32] Barner-Kowollik C., Russell G.T. (2009). Chain-length-dependent termination in radical polymerization: Subtle revolution in tackling a long-standing challenge. Prog. Polym. Sci..

[33] Buback M., Kurz C.H., Schmaltz C. (1998). Pressure dependence of propagation rate coefficients in free-radical homopolymerizations of methyl acrylate and dodecyl acrylate. Macromol. Chem. Phys..

[34] Barner-Kowollik C., Buback M., Egorov M., Fukuda T., Goto A., Olaj O.F., Russell G.T., Vana P., Yamada B., Zetterlund P.B. (2005). Critically evaluated termination rate coefficients for free-radical polymerization: Experimental methods. Prog. Polym. Sci..

[35] Ribelli T.G., Wahidur Rahaman S.M., Daran J.C., Krys P., Matyjaszewski K., Poli R. (2016). Effect of Ligand Structure on the Cu II –R OMRP Dormant Species and Its Consequences for Catalytic Radical Termination in ATRP. Macromolecules.

[36] Fantin M., Lorandi F., Ribelli T.G., Szczepaniak G., Enciso A.E., Fliedel C., Thevenin L., Isse A.A., Poli R., Matyjaszewski K. (2019). Impact of Organometallic Intermediates on Copper-Catalyzed Atom Transfer Radical Polymerization. Macromolecules.

