# Supplementary material for: ATRP of Methyl Acrylate by Continuous Feeding of Activators Giving Polymers with Predictable End-Group Fidelity
Source: Polymers (Basel). 2019 Jul 26;11(8):1238. doi: 10.3390/polym11081238 (PMC6724064; doi:10.3390/polym11081238)
Supplement: Supplementary file 1 [file polymers-11-01238-s001.pdf]

Supporting Information: ATRP of Methyl Acrylate by Continuous Feeding of Activators Giving Polymers with Predictable End-Group Fidelity

Yu Wang*

Department of Chemistry, University of Louisiana at Lafayette, Lafayette, LA, USA

E-mail: yuwang@louisiana.edu

Supporting Data

Derivation of Equation 4 in the main text:

$$\ln \frac{[\mathrm{M}]_0}{[\mathrm{M}]} = k_{\mathrm{p}}[\mathrm{R}^\bullet]t$$

$$\Delta[\mathrm{Br}]_{\mathrm{loss}} = [\mathrm{Cu(I)}]_{\mathrm{total}} = 2k_{\mathrm{t}}^{\mathrm{app}}[\mathrm{R}^\bullet]^2 t$$

$$k_t^{\mathrm{app}} = \frac{k_p^2 t[\mathrm{Cu(I)}]_{\mathrm{total}}}{2\ln^2 \frac{[\mathrm{M}]_0}{[\mathrm{M}]}}$$

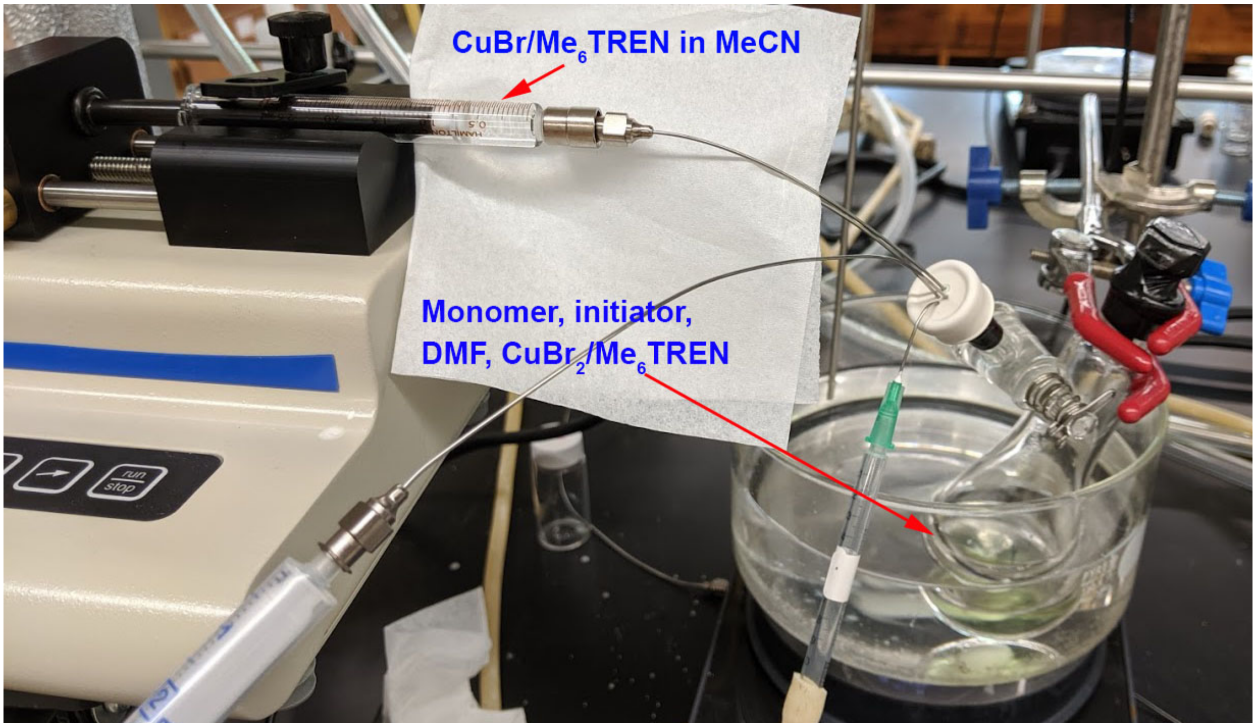

Figure S1: Experimental setup for ATRP by continuous feeding of activators.

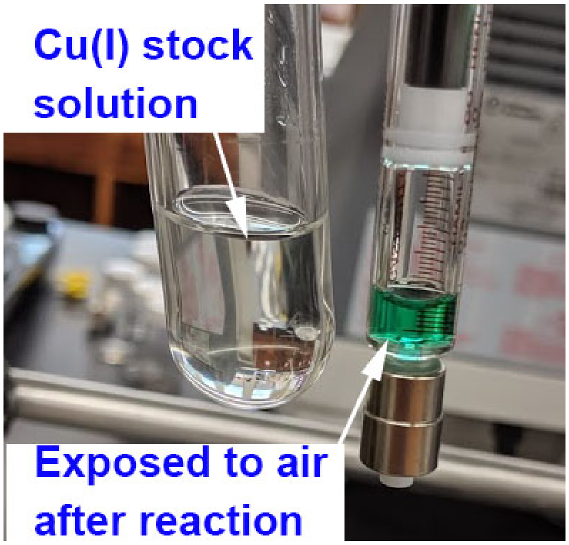

Figure S2: Color of the Cu(I) stock solution and when it was exposed to air after the reaction.

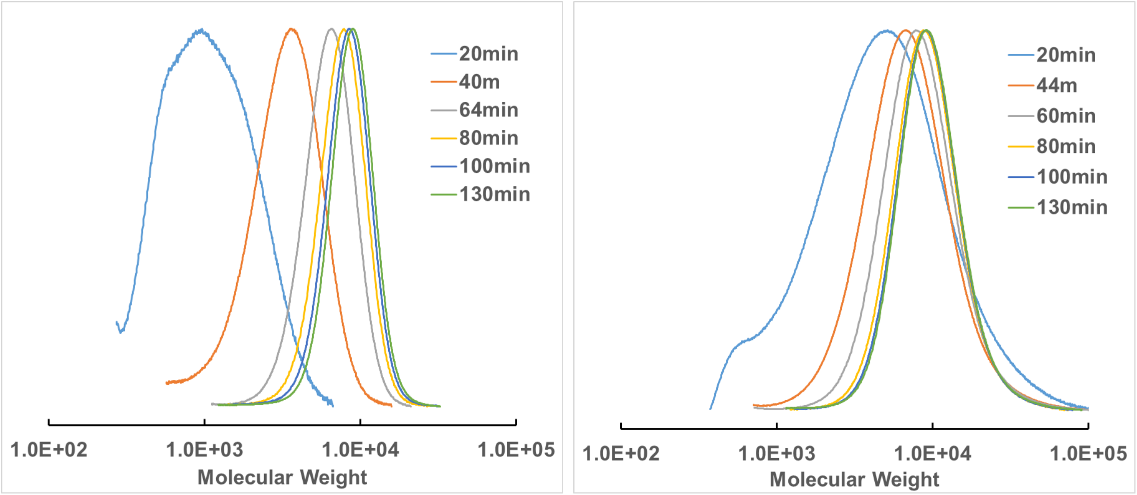

Figure S3: GPC curves for ATRP of MA by continuous feeding of Cu(I) activators at 60 °C with 100 mmol MA in 5 mL of DMF; 0.04 mmol CuBr/Me_6TREN in 2 mL of MeCN was added in 2 h (a) with initially-added Cu(II), $[MA]_0 : [MBrP]_0 : [CuBr_2]_0 : [Me_6TREN]_0 = 100 : 1 : 0.005 : 0.01$, and (b) without initially-added Cu(II), $[MA]_0 : [MBrP]_0 = 100 : 1$.

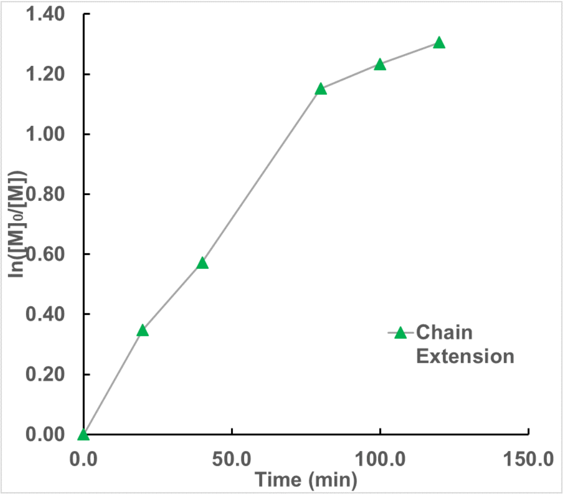

Figure S4: Kinetic plots of $\ln([M]_0/[M])$ vs. time for ATRP of MA by continuous feeding of Cu(I) activators for the chain extension with PMA_{43}−Br as the macroinitiator. $[MA]_0 : [PMA_{43}-Br]_0 : [CuBr_2]_0 : [Me_6TREN]_0 = 50 : 1 : 0.0025 : 0.005$ at 60 °C with 50 mmol MA in 5 mL of DMF; 0.04 mmol CuBr/Me_6TREN in 2 mL of MeCN was added in 2 h.
